# Presbyopia: What We Do Know and What We Do Not Know in 2022

**DOI:** 10.3390/jcm12030794

**Published:** 2023-01-19

**Authors:** Andrzej Grzybowski, Maciej Gawęcki

**Affiliations:** 1 Department of Ophthalmology, University of Warmia and Mazury, 10-719 Olsztyn, Poland; 2 Institute for Research in Ophthalmology, 60-554 Poznan, Poland; 3 Institute for Research in Ophthalmology, Foundation for Ophthalmology Development, 61-553 Poznan, Poland; 4 Dobry Wzrok Ophthalmological Clinic, 80-822 Gdansk, Poland

The subject of presbyopia has accompanied clinical ophthalmic practices around the world for centuries. Methods for the correction of presbyopia have evolved with the progress in applied optics, pharmacology and surgical techniques, including refractive surgery. Despite the availability of numerous options for presbyopia correction, none are devoid of drawbacks or inconveniences for the patients. As modern patients’ expectations are very high and diverse, there is a need to meet them with a choice of suitable methods for presbyopia correction to minimize the possible inconveniences. On the other hand, this sets high standards for ophthalmologists, that should possess current knowledge on state-of-the-art presbyopia correction methods, especially the side effects and complications.

The present Special Issue “Hot Topics in Presbyopia 2021” was thought of to respond to the need for constant updates on this subject. It contains a range of papers that discuss selected problems associated with presbyopia correction in modern ophthalmological practices. The subject discussed most intensively in this issue is the correction of presbyopia with intraocular lenses (IOLs). The employment of different optical designs in IOLs has a consequence in its clinical performance and elicitation of dysphotopsias [1]. Modern cataract surgery has faced a shift towards the implantation of EDOF IOLs instead of the traditionally used multifocal IOLs (mIOLs) due to the quality-of-vision problems that more frequently occur with multifocal lenses [2]. The optical features of different IOLs have to be evaluated in laboratory studies to precisely understand their clinical performance. This subject is investigated in the paper by Monatgud-Martinez et al. [3]. The authors performed an in vitro (optical bench) comparison of three diffractive–refractive presbyopia- correcting IOLs with different optical design: trifocal, EDOF and enhanced monofocal, and tested them with monochromatic and polychromatic light. They report the defocus curve and modular transfer function of each IOL under different lightning conditions, providing additional information on their chromatic aberration and its effect on clinical performance. The analogous polychromatic analysis for a refractive design-segmented EDOF IOL was presented by Garcia et al. [4]. The authors reported the in vitro performance of this IOL, revealing primarily its bifocality as responsible for the EDOF effect with just minor significance of chromatic aberrations. The issue also presents two clinical papers that reported the performance of premium IOLs in practice. Nováček et al. reported the differences in refractive and functional outcomes between two trifocal IOLs made from the same material but with different optical design [5]. Despite the technological similarities, differences in the optical construction of the IOLs resulted in variable performances according to the refraction outcome, contrast sensitivity and posterior capsule opacification. Cervantes-Coste et al. presented an interesting analysis of the influence of the pre-operative angle alfa and kappa on the post-operative functional outcomes after the implantation of a trifocal IOL [6]. In their analysis researchers concluded that measurements of the angle alpha as well as the higher order aberrations before surgery with multifocal IOLs can provide additional information for the patient’s selection for the procedure. Results of the interesting questionnaire evaluating the application of mIOLs in patients with retinal disorders are presented in the paper by Lee et al. [7]. Korean retinal specialists disapproved the use of mIOLs in patients with wet age-related macular degeneration, dry age-related macular degeneration with geographic atrophy, proliferative diabetic retinopathy, non-proliferative diabetic retinopathy with macular edema, previous macula-off retinal detachment, previous retinal vein occlusion, and epiretinal membrane. This report reflects the common opinion about the drawbacks of mIOLs in the presence of retinal damage, nevertheless this question needs further research and precise recommendations, that so far have not yet been formulated [8]. 

Every surgical procedure involving the anterior segment has the potential to worsen or cause a dry eye disease. Presbyopia surgical correction has been reviewed in a large analysis by Mikalauskiene et al. [9]. The authors performed a thorough search of the available literature and presented a synthesis on the classification, diagnostic criteria and prevalence of the disorder in the context of planned surgery. The set of practical tips for the pre-operative assessment and post-operative management of a patient with ocular surface problems is provided. 

Moreover, this issue contains a review on the pharmacological treatment of presbyopia [10]. Nowadays the subject is rarely analyzed because the surgical options for presbyopia have dominated the ophthalmological market [11]. Nevertheless, it must be emphasized, that 1.25% pilocarpine has recently been approved by the FDA for presbyopia treatment. Grzybowski and Ruamviboonsuk presented the role of the topically applied miotics and lens softeners as therapeutic agents for the treatment of presbyopia [10]. They also outlined the potential pathways for future research on topical drugs in presbyopia management. 

Articles presented in this Special Issue reflect the need for constant research on presbyopia management. They provide both our current knowledge and the imperfections of available technologies. Thus, we hope that this issue will help to elicit new ideas and stimulate the onset of further studies on the subject.
